# Thyroid Cancer in Childhood Cancer Survivors: Demographic, Clinical, Germline Genetic Characteristics, Treatment, and Outcome

**DOI:** 10.3390/jcm14020589

**Published:** 2025-01-17

**Authors:** Ulku Miray Yildirim, Rejin Kebudi, Ayça İribaş Çelik, Bülent Zülfikar, Abut Kebudi

**Affiliations:** 1 Department of Pediatric Hematology and Oncology, Oncology Institute, Istanbul University, 34093 Istanbul, Turkey; ulku-miray@hotmail.com (U.M.Y.); bulent.zulfikar@istanbulmedicare.com (B.Z.); 2 Department of Radiation Oncology, Medical Faculty, Istanbul University, 34093 Istanbul, Turkey; aycairibas@hotmail.com; 3 Department of Surgical Oncology, Medical Faculty, Okan University, 34947 Istanbul, Turkey; abutkebudi@gmail.com

**Keywords:** childhood cancer survivors, thyroid cancer, second malignant neoplasms, radiation therapy, genetic mutations

## Abstract

**Objective:** Childhood cancer survival rates have improved, but survivors face an increased risk of second malignant neoplasms (SMNs), particularly thyroid cancer. This study examines the demographic, clinical, genetic, and treatment characteristics of childhood cancer survivors who developed thyroid cancer as a second or third malignancy, emphasizing the importance of long-term surveillance. **Methods:** A retrospective review was conducted for childhood cancer survivors treated between 1990 and 2018 who later developed thyroid cancer as a second or third malignancy. Data on demographics, clinical characteristics, treatment, and outcomes were analyzed. **Results:** Among the 3204 childhood cancer survivors, 10 patients (6 female, 4 male) developed papillary thyroid carcinoma (PTC), a median of 9 years post-initial diagnosis. Radiation therapy, particularly to the head and neck, was commonly used. Genetic testing revealed mutations in the Cell Cycle CheckPoint Kinase 2 (CHEK2) and Adenomatous Polyposis Coli (APC) genes in four patients, possibly contributing to the increased risk. All were diagnosed through thyroid ultrasound and underwent total thyroidectomy, and three received radioactive iodine (RAI). No recurrences or deaths related to PTC occurred, with a median follow-up of 5.5 years after diagnosis. **Conclusions:** Radiation therapy, especially combined with chemotherapy, significantly increases the risk of thyroid cancer in childhood cancer survivors. Genetic predispositions also play a role. Lifelong thyroid cancer surveillance is essential, particularly for those who received radiation or chemotherapy. Further research is needed to refine surveillance strategies and better understand genetic factors that influence thyroid cancer risk. Early detection and ongoing monitoring are critical for improving long-term outcomes.

## 1. Introduction

Over the past few decades, significant advancements in pediatric oncology have led to a marked increase in the survival rates of children diagnosed with cancer. These improvements can be attributed to enhanced access to innovative treatments, better supportive care, and advancements in early diagnosis, which have collectively improved the outcomes of childhood cancer patients [1,2]. As survival rates continue to rise, the long-term health effects of cancer treatments have become an increasingly critical focus of research and clinical practice. Childhood cancer survivors often experience a range of late effects, which can include physical, emotional, and psychological consequences resulting from their treatments. Among these late effects, the development of second malignant neoplasms (SMNs) has emerged as one of the most significant concerns for survivors of childhood cancers [3]. Among the various types of SMNs that can develop, thyroid cancer has been identified as one of the most common and clinically relevant malignancies in this group of patients [4,5]. The risk of developing thyroid cancer as an SMN is well-documented, particularly for those individuals who have undergone radiation therapy as part of their treatment regimen for the primary malignancy. However, this risk is not limited to those who have received radiation; patients who have been treated with chemotherapy, particularly with certain chemotherapy agents, such as alkylating agents or anthracyclines, or with targeted therapies like I-131 MIBG, are also at increased risk of developing secondary thyroid cancer. The relationship between radiation exposure and the development of thyroid cancer has been extensively studied, with research demonstrating a dose-dependent association between the amount of radiation received and the risk of thyroid malignancy [6]. In addition to radiation exposure, genetic predisposition plays an important role in the development of secondary thyroid cancers. Certain inherited genetic mutations or syndromes may increase the susceptibility of childhood cancer survivors to developing thyroid cancer [7].

In this context, this study aims to assess patients who developed thyroid cancer as a second/third malignancy among childhood cancer survivors in a single center and emphasize the importance of long-term monitoring for this population.

## 2. Materials and Methods

We conducted a retrospective review of medical records for childhood cancer survivors treated between 1990 and 2018 and followed up for at least five years at the pediatric oncology clinic of a referral hospital who subsequently developed thyroid cancer as a second or third malignancy. Demographic, clinical, and radiological characteristics, as well as treatment and outcome of the patients, were evaluated. Follow-up duration was recorded from the time of diagnosis of thyroid cancer to November 2024 or until the last visit. Germline genetic testing for predisposing genes was investigated by next-generation sequencing in four patients, in three of whom there was a third subsequent malignancy. This study is approved by the institutional ethical review board.

## 3. Results

Among the 3204 children treated for cancer between 1990 and 2018, 10 patients (six female, four male) with a median age of 7.5 years (range 12 months to 16 years) at diagnosis developed thyroid cancer at a median of 9 years (range 4 to 19 years) after their initial cancer diagnosis. All cases were histologically classified as papillary thyroid carcinoma (PTC). Patient characteristics are summarized in Table 1.

All patients had received chemotherapy. Eight of the ten patients had received radiotherapy, seven to the head and neck region.

The primary diagnosis for the seven patients who received radiotherapy that included the head and neck region were brain tumors in four (three patients with medulloblastomas and one with pineoblastoma), Hodgkin lymphoma in two, and rhabdomyosarcoma of the head and neck region in one patient.

The patient with rhabdomyosarcoma of the head and neck developed thyroid carcinoma 16 years from diagnosis. The tumor was not within the radiation site, but she may have been affected by the scattered radiation. She received radioactive RAI and is in remission for thyroid cancer. She further developed breast cancer 28 years after diagnosis. She received surgery, radiotherapy, and chemotherapy for breast cancer. Molecular studies identified a germline mutation of *CHEK2.*

In a patient diagnosed with medulloblastoma who developed thyroid carcinoma 7 years later, a malignant mesenchymal tumor of head and neck origin was diagnosed 12 years after the initial cancer diagnosis. Molecular studies identified a germline mutation in *APC*.

A patient with sarcoma of the ovary had received whole abdomen radiotherapy (WART) and was found to have a germline *DICER1* mutation. She underwent surgery for a pancreatic pseudocyst two years after completing treatment and was monitored for microcysts and hypoechoic nodules in both thyroid glands. Initial biopsy (performed 5 years after the first malignancy diagnosis) results were classified as indeterminate atypia, but follow-up revealed nodule growth, leading to a repeat biopsy (performed 7 years after the first malignancy diagnosis) that confirmed papillary carcinoma (multifocal papillary carcinoma with follicular subtype, no capsule invasion).

The patient with pinealoblastoma was also tested for a hereditary cancer panel, including DICER1; however, a predisposing condition could not be detected.

The other two patients had received chemotherapy and surgery and no radiotherapy. These two patients, one with infant neuroblastoma and another with osteosarcoma, developed renal cell carcinoma as a second malignancy (9 and 14 years after the initial malignancy diagnosis, respectively) prior to their thyroid cancer diagnosis. The patient with neuroblastoma was investigated for a hereditary cancer panel with next-generation sequencing (NGS); however, a predisposing condition could not be detected. In the patient diagnosed with osteosarcoma, genetic testing could not be performed.

None of the patients included in the study had a family history of thyroid cancer.

All patients were diagnosed by thyroid ultrasound during surveillance and underwent total thyroidectomy; three patients received radioactive iodine (RAI). There were no recurrences or deaths attributed to PTC. One patient with medulloblastoma died due to an accident. The remaining nine patients are alive, with a median follow-up of 5.5 years (range 0.5 to 15 years) post-thyroid cancer diagnosis.

## 4. Discussion

Survival rates for childhood cancers have markedly improved over the past few decades, leading to a growing population of survivors. However, the treatment modalities—such as chemotherapy and radiotherapy—are associated with significant long-term side effects. The emergence of SMNs remains one of the leading causes of treatment-related mortality in cancer survivors, warranting increased attention [6]. Thyroid cancer, identified as one of the most common SMNs, affects approximately up to 10% of childhood cancer survivors [8]. Consistent with existing literature, our findings indicate that all patients in this cohort were diagnosed with papillary thyroid carcinoma [9]. The risk of developing SMNs varies based on several factors, including age at primary diagnosis, treatment modalities received, and genetic predisposition [10].

Radiation therapy, particularly, is a well-documented risk factor for secondary thyroid cancer (STC) among childhood cancer survivors. The latency period for the development of secondary primary tumors after radiotherapy has been previously reported as 20–30 years [11]. However, Clement et al. [12], summarizing the results of 16 studies, have reported the latency period as 10–20 years. The same study mentions that STC can be observed as early as 4.2 years after radiation therapy. Patients included in our study have a median time of 9 years—with the earliest of 4 years (range 4 to 19 years) from the primary cancer diagnosis to the diagnosis of thyroid cancer. The International Late Effects of Childhood Cancer Guideline Harmonization Group, in collaboration with the PanCareSurFup Consortium, recommends initiating surveillance for STC at the latest five years after radiation therapy and extending the follow-up period appropriately [12].

Several studies have been conducted to investigate the relationship between radiation dose and the development of thyroid cancer. The Childhood Cancer Survivor Study analyzed 119 cases of STC among 12,547 childhood cancer survivors diagnosed between 1970 and 1986 and reported a linear increase in the risk of developing STC correlated with the dose of radiation therapy (RT) received, with the highest relative risk observed in patients exposed to approximately 20 Gy of radiation. Specifically, a relative risk peak of 14.6 was noted for those receiving this dose compared to those who did not receive radiation. However, at radiation doses exceeding 20 Gy, the relationship between dose and risk appeared to diminish [13]. This study also evaluated the risk associated with chemotherapy and the potential combined effects of RT and chemotherapy. They found that treatment with alkylating agents was linked to a 2.4-fold increased risk of developing secondary thyroid cancer among survivors who had received RT doses of 20 Gy or less. Notably, this increased risk was not observed in patients who received higher doses of RT. This is thought to be due to the cell-killing effect of high radiation doses potentially obscuring the effects of chemotherapy. This suggests that while RT remains a significant risk factor for thyroid cancer, certain chemotherapy agents may also contribute to this risk, highlighting the importance of considering both treatment modalities when assessing long-term cancer risks in childhood cancer survivors [14]. Additionally, although radiation exposure is the most significant risk factor, there are cases of thyroid cancer occurring at radiation doses below 1 Gy and above 40 Gy, indicating that a universally safe radiation threshold has yet to be established [15].

Veiga et al. (2012) [14] reported that while radiation therapy was a well-established risk factor, certain chemotherapy agents, particularly alkylating agents, and anthracyclines, were associated with an increased risk of thyroid cancer, even in patients who had not received radiotherapy. Black et al. [16] also discussed cases where patients developed thyroid cancer without prior radiation exposure in their series of 18 STC cases (one patient had not received any radiation therapy, and two patients had received radiation therapy to areas distant from the thyroid). Our findings also included two patients (osteosarcoma and neuroblastoma) who developed thyroid carcinoma after chemotherapy without preceding radiation therapy. The patient with ovarian sarcoma received radiotherapy to the whole abdomen, which is a distant site. Although the patient with rhabdomyosarcoma of the head and neck received radiotherapy, the thyroid carcinoma was not within the radiation field. These findings suggested that chemotherapy could contribute to the development of secondary thyroid malignancies, highlighting the importance of ongoing surveillance for thyroid cancer in this population, regardless of prior radiation exposure.

The reduction in radiation-related thyroid cancer risk with increasing age at exposure has been demonstrated in several studies [17,18]. Studies conducted with atomic bomb survivors, a rare and unique population in which radiation risks have been assessed across a wide range of exposure ages, have shown a decrease in radiation risk with increasing age at exposure. Additionally, no cases of radiation-related thyroid cancer have been observed in the population aged 20–39 years in this cohort [19]. In the study by Taylor et al. (2009) [20], the authors investigated the risk of thyroid cancer in survivors of childhood cancer as part of the British Childhood Cancer Survivor Study. The results indicated that the age at which patients received radiation therapy significantly influenced the risk of developing thyroid cancer. Specifically, younger patients, particularly those under the age of 5 at the time of radiation exposure, exhibited a markedly higher risk compared to older children. The study also highlighted that as the age at exposure increased, the relative risk of developing thyroid cancer decreased. These findings emphasize the critical importance of considering age at treatment when assessing long-term cancer risks in childhood cancer survivors and suggest a need for tailored follow-up strategies based on age and treatment history.

(131) I-Metaiodobenzylguanidine (MIBG), a guanidine derivative taken up by a norepinephrine reuptake protein located on the surface of most NBL cells, is an alternative therapeutic approach as targeted radiotherapy in the treatment of neuroblastoma. Thyroid disorders, including thyroid carcinoma, have been observed in up to half of the patients 1.4 years after treatment with (131) I-MIBG for neuroblastoma in children, even with the administration of potassium iodide. The first two documented cases of differentiated thyroid carcinoma that developed in children following treatment with (131) I-MIBG for neuroblastoma presented with thyroid malignancies years after MIBG therapy were previously described. To prevent thyroidal iodine uptake, potassium iodide (KI) prophylaxis is administered to children treated with 131I-MIBG, thus diluting the free circulating radio-iodine (derived from 131I-MIBG) and reducing the amount of iodine taken up by the thyroid gland. Despite these precautions, hypothyroidism, thyroid nodules, and thyroid cancer have been reported as various forms of thyroid damage following 131I-MIBG treatment. These damages are thought to be caused by the thyroidal uptake of free circulating radio-iodine [21,22].

In this context, to inform the clinical management of childhood, adolescent, and young adult cancer (CAYAC) survivors at elevated risk for differentiated thyroid cancer (DTC), the International Late Effects of Childhood Cancer Guideline Harmonization Group (IGHG) convened an expert panel tasked with reviewing and synthesizing all available evidence on the risk factors for DTC, as well as evaluating various strategies for the screening of subclinical DTC. According to IGHG, CAYAC survivors who have received radiation therapy involving the thyroid gland or therapeutic 131 I-MIBG are at elevated risk for DTC, and it is recommended to utilize thyroid ultrasonography as a screening modality for the detection of thyroid abnormalities. It is also considered reasonable to initiate surveillance for DTC 5 years following radiation therapy involving the thyroid gland or therapeutic 131I-MIBG and repeat thyroid ultrasonography every 3–5 years. Although no specific guidance is provided regarding the duration of ongoing surveillance, it may be continued throughout life due to the potential for late-onset occurrences [12].

Moreover, our study highlights the importance of considering genetic predispositions in patients developing secondary malignancies. In our study, one of the patients developed breast cancer as a third tumor, and a *CHEK2* mutation was detected. Cell cycle checkpoint kinase 2 (CHEK2) is a crucial element in the DNA damage response pathway and is linked to an increased risk of various cancers, including rhabdomyosarcoma and breast cancer. The CHEK2 gene encodes a protein kinase involved in the DNA damage response, cell cycle checkpoint regulation, and the maintenance of genomic stability. Mutations in CHEK2, particularly those that result in loss of function, compromise these key cellular functions, leading to an increased susceptibility to the development of malignancies, including thyroid cancer. Specifically, CHEK2 mutations can impair the cell’s ability to detect DNA damage and halt cell division, allowing cells with genetic errors to proliferate uncontrollably. This dysfunction may contribute to carcinogenesis by facilitating tumor initiation and progression. There have been reports on the increased risk for thyroid cancers with *CHEK2* mutations [23,24,25,26]. However, the available data are limited, which makes them insufficient to support definitive recommendations for surveillance [27]. One of the co-authors (RK) had a patient with relapsed ALL, whose mother was diagnosed with papillary thyroid carcinoma as an adult, and both the mother and child were found to have a germline CHEK2 mutation. An APC mutation was detected in a patient with a primary diagnosis of medulloblastoma who developed a malignant mesenchymal tumor as a tertiary malignancy in our study cohort. The APC gene plays a critical role in the Wnt signaling pathway, which regulates cell growth, differentiation, and adhesion. Mutations in APC often lead to the dysregulation of this pathway, resulting in the accumulation of β-catenin in the nucleus, where it can activate the transcription of genes involved in cell proliferation. In thyroid cells, aberrant Wnt signaling due to APC mutations may contribute to uncontrolled cellular growth, driving the development of thyroid carcinomas, including papillary thyroid carcinoma [28]. Individuals with familial adenomatous polyposis resulting from pathogenic germline mutations in the APC gene face a significantly elevated risk of developing papillary thyroid cancer besides colon polyps, and annual thyroid ultrasonography is recommended for thyroid cancer screening; however, there is currently no established consensus on the appropriate age to begin screening [7,29]. Additionally, given the roles of CHEK2 and APC in key cellular pathways, targeted therapies that aim to restore normal function in these pathways could hold promise for the management of secondary thyroid cancer in childhood cancer survivors [30].

In the patient diagnosed with ovarian sarcoma, the atypical diagnosis compared to common ovarian tumors in this age group, along with the presence of air cysts in the lungs and microcysts in the thyroid lobes, raised suspicion for DICER1 syndrome, which is a rare hereditary cancer predisposition syndrome associated with a wide range of multi-organ neoplastic and non-neoplastic conditions [31]. DICER1 is generally regarded as either a tumor suppressor gene when loss-of-function mutations occur or as an oncogene when gain-of-function mutations are present. In individuals diagnosed with DICER1 syndrome, the majority of tumors develop in those who inherit a DICER1 mutation and also acquire an additional somatic missense mutation in the 5′ “hot-spot” codons of the RNAse IIIb domain. This combined genetic alteration leads to the activation of the PI3K/AKT/mTOR signaling pathway [32]. Epidemiological analysis of a large cohort of individuals with one or more DICER1-associated conditions, compared to controls, reveals that by 20 years of age, the cumulative incidence of multinodular goiter or a history of thyroidectomy is 32% in women and 13% in men. Individuals with DICER1 mutations have a 16- to 24-fold increased risk of developing thyroid cancer [33]. DICER1 mutation may also be a predisposing factor in pinealoblastomas. A genetic predisposition could not be found in our patient with pinealoblastoma. Consensus guidelines developed according to the data from the International Pleuropulmonary Blastoma (PPB) Registry recommend that individuals with DICER1 pathogenic variants and DICER1-associated conditions, particularly children, should be counseled on their increased risk of thyroid nodules and thyroid cancer. It is recommended that children undergo baseline thyroid ultrasonography at around 8 years of age, followed by regular screenings every 2 to 3 years. For those who have received chemotherapy or radiation for non-thyroid malignancies, thyroid ultrasonography should be performed at diagnosis and annually for the first 5 years, with the frequency reduced to every 2 to 3 years if no abnormalities are detected [34].

When evaluating individuals with thyroid cancer, in addition to the genetic alterations identified in our study, the presence of hereditary conditions that could represent familial backgrounds, such as multiple endocrine neoplasia type 2 (MEN 2) in medullary thyroid cancer and Li-Fraumeni syndrome, Cowden syndrome, Carney complex, and Werner syndrome in non-medullary thyroid cancers, should also be considered [35].

Future Research Directions:

To further improve the long-term health and quality of life for childhood cancer survivors, future research should focus on several key areas. First, refining genetic screening protocols is essential for identifying individuals at increased risk for developing secondary thyroid cancer. Targeted genetic testing for mutations such as those in DICER1, CHEK2, and APC could help pinpoint high-risk individuals, facilitating earlier intervention and more personalized surveillance strategies. The integration of genetic testing into routine clinical care, alongside radiation and chemotherapy history, could serve as a vital tool for predicting cancer risk in childhood cancer survivors.

Moreover, research should explore the potential for preventative interventions in these high-risk populations. This could include the development of chemopreventive strategies, such as the use of drugs to target specific genetic defects or pathways known to be disrupted in thyroid cancer. Additionally, studies evaluating the effectiveness of early and intensive surveillance methods, including advanced imaging techniques and molecular markers, will be critical in improving early detection and treatment outcomes.

Further large-scale, multicenter studies are necessary to understand the complex interplay between genetic factors, treatment modalities, and environmental exposures that contribute to thyroid cancer risk in childhood cancer survivors. These studies could lead to the identification of new biomarkers for early detection and provide evidence for more tailored treatment and prevention protocols.

In summary, enhancing awareness of the genetic and environmental factors that influence thyroid cancer risk in survivors, combined with proactive monitoring strategies, will be key to addressing the health needs of this vulnerable population. By focusing on these areas, we can significantly improve the outcomes and long-term well-being of childhood cancer survivors.

## Figures and Tables

**Table 1:** Patient characteristics.

Patient/Gender	Age at Diagnosis of First Malignancy (Years)	Diagnosis of First Malignancy	Treatment for First Malignancy	Time to Diagnosis of Thyroid Cancer After the Initial Cancer Diagnosis (Years)	Status at Last Follow-Up
Patient 1/F *	9	Rhabdomyosarcoma (head and neck)	CT, RT	16	Alive with NED
Patient 2/M **	1	Neuroblastoma	CT, S	19	Alive with NED
Patient 3/M ***	16	Osteosarcoma	CT, S	18	Alive with NED
Patient 4/M	10	Hodgkin Lymphoma	CT, RT	4	Alive with NED
Patient 5/F	10	Pinealoblastoma	S, RT, CT	10	Alive with NED
Patient 6/F	7	Medulloblastoma	S, RT, CT	9	Alive with NED
Patient 7/F ****	7	Medulloblastoma	S, RT	7	Alive with D
Patient 8/F	5	Medulloblastoma	S, RT, CT	9	EX
Patient 9/M	8	Hodgkin Lymphoma	CT, RT	5	Alive with NED
Patient 10/F	4	Sarcoma (Ovarian)	S, CT, RT	7	Alive with NED

CT, chemotherapy; D, disease; F, female; M, male; NED, no evidence of disease; RT, radiation therapy; S, surgery; EX, excitus. * The patient was diagnosed with breast cancer at the age of 39 as a third malignancy and was found to have a Cell Cycle Checkpoint Kinase 2 (CHEK2) mutation. ** The patient was diagnosed with renal cell carcinoma at the age of nine as a second malignancy. *** The patient was diagnosed with renal cell carcinoma at the age of 30 as a second malignancy. **** The patient was diagnosed with soft tissue sarcoma at the age of 19 as a third malignancy and found to have Adenomatous Polyposis Coli (APC) mutation.

## Data Availability

The data presented in this study are available upon reasonable request from the corresponding author. The data are not publicly available due to ethical commitments for sensitive patient information.
